# Proteome and allergenome of the European house dust mite *Dermatophagoides pteronyssinus*

**DOI:** 10.1371/journal.pone.0216171

**Published:** 2019-05-01

**Authors:** Rose Waldron, Jamie McGowan, Natasha Gordon, Charley McCarthy, E. Bruce Mitchell, David A. Fitzpatrick

**Affiliations:** 1 Department of Biology, National University of Ireland Maynooth, Co. Kildare, Ireland; 2 Airmid Healthgroup Ltd., Trinity Enterprise Campus, Dublin, Ireland; 3 Human Health Research Institute, Maynooth University, Maynooth, Co. Kildare, Ireland; Onderstepoort Veterinary Institute, SOUTH AFRICA

## Abstract

The European house dust mite *Dermatophagoides pteronyssinus* is of significant medical importance as it is a major elicitor of allergic illnesses. In this analysis we have undertaken comprehensive bioinformatic and proteomic examination of *Dermatophagoides pteronyssinus* airmid, identified 12,530 predicted proteins and validated the expression of 4,002 proteins. Examination of homology between predicted proteins and allergens from other species revealed as much as 2.6% of the *D*. *pteronyssinus airmid* proteins may cause an allergenic response. Many of the potential allergens have evidence for expression (*n* = 259) and excretion (*n* = 161) making them interesting targets for future allergen studies. Comparative proteomic analysis of mite body and spent growth medium facilitated qualitative assessment of mite group allergen localisation. Protein extracts from house dust contain a substantial number of uncharacterised *D*. *pteronyssinus* proteins in addition to known and putative allergens. Novel *D*. *pteronyssinus* proteins were identified to be highly abundant both in house dust and laboratory cultures and included numerous carbohydrate active enzymes that may be involved in cuticle remodelling, bacteriophagy or mycophagy. These data may have clinical applications in the development of allergen-specific immunotherapy that mimic natural exposure. Using a phylogenomic approach utilising a supermatrix and supertree methodologies we also show that *D*. *pteronyssinus* is more closely related to *Euroglyphus maynei* than *Dermatophagoides farinae*.

## Introduction

House dust mites (HDM) are the most prevalent source of indoor allergens worldwide, with 1–2% of the total population experiencing an allergic response in their presence [1]. HDM allergens are major causative agents in the pathogenesis of asthma, allergic rhinitis and atopic dermatitis [2]. Protease allergens disrupt the epithelial barrier and activate immune cells resulting in the production of large amounts of proinflammatory cytokines [3, 4]. Sero-dominant allergens; Der p 1and Der p 2 account for 50–60% of IgE reactivity in individuals tested [5]. Allergenic protein families represent only 2% of all protein families. Allergenicity and cross-reactivity is linked to the allergen family rather than allergen source [6]. Use of publicly available allergen databases to query newly sequenced genomes for the presence of potentially allergenic or cross-reactive proteins has enormous potential in identifying new allergens.

HDM allergens are either located within the mite body or in faecal particles. Current knowledge of mite allergen localisation is limited [7] and may be improved by employing comparative proteomics to study the mite body and spent culture media proteins of *D*. *pteronyssinus*. Proteins present in HDM faeces are of particular importance as faecal particles are inhaled deep into the lungs, due to their small size [4, 8, 9]. Very little is known about which HDM components are present in house dust or inhalable air, and assessment is limited to allergens for which there are ELISAs [10]. Therefore, it is our belief that characterising *D*. *pteronyssinus* proteins present in house dust could potentially yield much needed insights into allergens present in house dust.

Previous studies have shown that approximately 50% of all European homes contain HDM [11]. Therefore, evidenced based biocontrol strategies are needed to curtail HDM populations in homes. A Cochrane review of commonly used physical, chemical and combined physical chemical HDM control strategies has shown no clinical benefit or evidence that these control measures can reduce exposure to HDMs, their allergens or the severity/frequency of asthma symptoms [12]. Reducing humidity within the home has been proposed as a means of constraining HDM populations and limiting allergen production [13]. However, transient exposure to moist air allows for long term survival and reproduction indicating HDM may employ mechanisms to resist desiccation [14]. Therefore, genomic and proteomic characterisation of *D*. *pteronyssinus* has the potential to reveal biochemical pathways that could be exploited in future biocontrol strategies. These “Omic” approaches have accelerated digestive enzyme discovery [15], enabling *in silico* prediction of biochemical activities coupled with measurement of gene or protein expression. Potent enzymes are excreted by HDM into their surroundings as a by-product of their digestive processes, therefore the presence of putative enzymes in faeces is a strong indicator of a digestive function [16]. Surveying the predicted proteome of *D*. *pteronyssinus airmid* and subsequent proteomic examination of enzyme expression and localisation could identify new enzymes utilised in nutrient acquisition.

Here we describe the proteome of the European HDM *D*. *pteronyssinus* using a strain of mite housed at airmid healthgroup ltd. We have analysed the proteome in an attempt to elucidate the phylogenetic relationships between different species of HDMs and determined the localisation of allergenic components and enzymes involved in nutrient acquisition. The predicted proteome provides the basis to further understand the reported cross-reactivity between HDM and phylogenetically distinct species. We have examined and report the *D*. *pteronyssinus airmid* predicted proteome, mite body proteome and excretome with reference to a wild-type proteome as a means of (i) identifying potentially allergenic proteins, (ii) inferring localisation of allergenic and potentially allergenic molecules, and (iii) identifying proteins involved in key physiological processes.

## Materials and methods

### Genomic data

To construct and compare the phylogenetic relationships between the Acari, 12 genomes were downloaded from the NCBI database for use in the analyses (S1 Table). Two Arachnid outgroups namely the Arizona bark scorpion (*Centruroides sculpturatus*) and the American house spider (*Parasteatoda tepidariorum*) were also downloaded. Assembly completeness of each genome was assessed using BUSCO v3 (Benchmarking Universal Single-Copy Ortholog) [17] with the Arthropoda dataset. Comparative analysis of *D*. *pteronyssinus* genome assemblies were conducted against previously published *D*. *pteronyssinus* strains [18, 19].

### Phylogenetic analysis

Phylogenomic analysis of *D*. *pteronyssinus* was undertaken with reference to 11 other species (subclass Acari), consisting of six Parasitiformes and five Acariformes (S1 Table). Supermatrix and supertree phylogenomic methods were employed to infer the evolutionary relationships. Suitable phylogenetic markers were selected by locating single copy orthologs (from BUSCO analysis above) in the Acari and outgroup genomes. Single copy orthologs that were ubiquitously present (*n* = 111) were aligned using MUSCLE [20]. Individual gene families alignments were subsequently concatenated to yield a supermatrix 77,878 amino acids in length. This supermatrix was used to reconstruct a maximum likelihood phylogenomic species tree using RAxML [21] utilising the LG+G+I+F model as selected by ProtTest [22], branch supports were determined using 100 bootstrap replicates.

Single copy ortholog families, present in at least four species (*n* = 2,796), were identified and individually aligned using MUSCLE. Subsequent phylogenies were generated using FastTree [23]. A supertree was constructed using the matrix representation with parsimony (MRP) method implemented in Clann [24] using the 2,796 gene trees as input with 100 bootstrap replicates. The resultant phylogeny was visualised and annotated using the Interactive Tree of Life (iTOL) [25].

### Proteo-genomic analysis of assembly completeness

Proteo-genomic software Peppy [26] was used to generate peptide databases from three *D*. *pteronyssinus* assemblies [18, 19, 27]. LC-MS/MS spectra derived from proteomic experimentation on *D*. *pteronyssinus airmid* were searched against the six-frame translated genomes (maximum FDR 0.01; precursor tolerance 2000; fragment tolerance 300; digestion rules–cleavage acid R & K, missed cleavages 1; static mods -mod C 57.021464). For completeness and comparative purposes, the spectra were searched against the corresponding translated predicted protein-coding genes for each assembly. Output files were filtered to locate unique peptides and corresponding genomic locations.

### Annotation of predicted proteome

Annotation of predicted proteins was achieved using BLAST2GO Version 5.0 [28] to sequentially search SwissProt (Downloaded; 14/01/2018) then NCBInr (Downloaded; 30/08/2017) database. Gene Ontology (GO) terms were assigned to predicted proteins (GO cut off 55, GO weight 5, E value hit filter E^-06^ and default computational evidence codes). InterPro Scan was used to identify; families, domains, sites and repeats in predicted proteins (CCD, HAMAP, HMMPanther, HMMpfam, FPrintscan, BLASTPromDom) and performed secretion peptide prediction using SignalP ver4.0 in parallel. Mapping feature facilitated mapping of GO terms to enzyme codes.

### *D*. *pteronyssinus* specific proteins

*D*. *pteronyssinus* predicted proteins without BLASTp homology (*n* = 3,906) to proteins in NCBInr/SwissProt were searched (tBLASTn; E value ≤ E^-05^) against Acari genomes for presence of homologs in closely related species (S1 Table). Predicted proteins without significant homology were considered *D*. *pteronyssinus-*specific proteins (*n* = 1,848) and were then searched against other the other two available *D*. *pteronyssinus* genome assemblies [18, 19]. Proteins without homology to predicted proteins in these assemblies were considered *D*. *pteronyssinus airmid-*specific proteins.

### Identification of LEA-like proteins

LEA homologs were identified by performing BLASTp (E-value ≤ 1E^-05^) searches [29] against the Late Embryogenesis Abundant Proteins Database (LEAPdb) [30].

### Mite group allergen orthologs

The majority of mite allergens identified to date have been assigned to groups (Group 1–33) in accordance with their order of discovery [7]. Chan *et al* (2015) reported a further seven non-chronological allergens for *D. farinae [31].* Mite group allergen (MGA) orthologs were identified by performing BLASTp searches of query FASTA files containing MGA from *D*. *pteronyssinus* & *D*. *farinae* (when no other sequences were available for *D*. *pteronyssinus*) against the Acari genomes utilised in this study (S1 Table). Allergen orthologs had to satisfy the following criteria; have reciprocal best BALST hits (RBH) with an allergen (E-value ≤ 1E^-05^) and a minimum alignment length of 80 amino acids with an identity ≥ 35% in accordance with FAO/WHO guidelines [32]. Allergens that had a RBH but did not meet the alignment and identity criteria were considered RBH homologs, these were included in the sequence similarity network and visualised using Gephi [33]. Each protein was represented by a node, two proteins were connected by an undirected edge if they were homologous (BLASTp ≥ 1E^-05^).

### Allergens and predicted allergens

*D*. *pteronyssinus airmid* MGA and non-chronological allergens were located by performing a local BLASTp (E-value ≤ 1E-03) search of the *D*. *pteronyssinus* predicted proteome against the FASTA sequence file containing the MGAs located above. The match with the smallest E-value was chosen as designated MGA. These sequences were annotated as “Der p1 Allergen” or Der f 22 like-allergen etc. for *D*. *farinae* based BLAST hits. Subsequent BLASTp hits were considered MGA homologs and were assessed for potential cross-reactivity with MGA in accordance with FAO/WHO guidelines [32]. Sequence similarity between known allergens (Uniprot “allergenome”) and *D*. *pteronyssinus airmid* predicted proteins was assessed in accordance with FAO/WHO guidelines [32].

### *D*. *pteronyssinus* airmid culture

*D*. *pteronyssinus airmid* were obtained from cultures housed at airmid healthgroup ltd (Dublin, Ireland) and maintained on diet composed of dried porcine liver and yeast, house dust mite maximal media (HDMMM, airmid healthgroup ltd, Ireland) at 75% relative humidity and 25°C.

### Harvest of mites and spent culture medium

Mites from replicate cultures (*n* = 5) were separated from spent culture medium (SM) by sieving, and saline floatation method [16], washed with distilled water and surface-sterilised by submersion in 70% ethanol (3 min) followed by washing with sterile distilled water. An average of 10.8 mites were present per mg of spent medium. Mites were then snap frozen in liquid nitrogen, and lyophilised. SM (*n* = 5) was divided into aliquots (200 mg) and stored at -70°C prior to protein extraction.

### Protein extraction

Lyophilised mite bodies (MB) were ground to a fine powder. Proteins were extracted from MB (25 mg) by addition of 500 μl lysis buffer and quantified according to methods described by Owens *et al* (2015) [34]. Proteins from spent culture medium (SM, *n* = 5) were extracted by addition of glass beads (50 mg; 0.5 mm BioSpec Products) and 1000 μl lysis buffer [34], followed by bead-beating (30 Hz; 5 min; MM300, Retsch®). MB (*n* = 2) and SM lysates (*n* = 2) were pooled for gel filtration (one biological replicate). Protein lysates for shotgun proteomic analysis (*n* = 4) were normalised (MB; 0.4 mg/ml, SM 0.3 mg/ml), then prepared for digestion according to Owens *et al* (2015) [34].

### Culture media contaminants

Proteins deriving from HDMMM (200 mg) were extracted (*n* = 1) and prepared for shotgun proteomic analysis as per methods for spent growth culture medium.

### House dust protein extracts

Der p 1 positive (0.2–16.94 μg Der p 1 per gram house dust) house dust protein extracts (*n* = 21) were provided by airmid healthgroup ltd (Dublin, Ireland).

### Gel filtration chromatography

Gel filtration chromatography was carried out using an ÄKTA purifier coupled with a Superdex 200 10/300 GL column (GE Healthcare, Germany), equilibrated in PBS. Filtered specimens (0.22 μm), were injected (500 μl) and separated (flow rate 0.4 ml/min) with absorbance monitored at 215, 254 and 280 nm. Fractions were collected (between ~ 6 and 32 ml) and stored at -20°C until further analysis.

### Proteolytic digestion of protein extracts for proteomic analysis

Specimens for proteomic analysis were prepared for LC-MS/MS as described by Owens *et al* (2015) [34].

### Nano-flow liquid chromatography electro-spray ionization tandem mass spectrometry (LC-MS/MS) analysis

Peptide mixtures were analysed using a Thermo Fisher Q-Exactive mass spectrometer coupled to a Dionex RSLC nano for LC-MS/MS analysis. LC gradients operated from 3–40% acetonitrile over 40 min, with data collection using a Top15 method for MS/MS scans [35].

### Representative proteome

LC-MS/MS spectra obtained from proteomic analysis of *D*. *pteronyssinus airmid* were randomised into 5 groups (14–20 files each). Spectra were searched using Sequest HT engine within Proteome Discoverer (Version 1.4) against *D*. *pteronyssinus airmid* predicted proteome (peptide filters; set to medium peptide confidence and protein filters; set to two peptides per protein). Protein molecular weight and pI for predicted proteome and representative proteome were calculated using JVirGel [36].

### MaxQuant and perseus data analysis

Protein identification and label free quantitative (LFQ) analysis was conducted using MaxQuant (Version 1.6.1.0; http://maxquant.org/), statistical analysis of MaxQuant output data was performed by Perseus (Version 1.6.2.2) as described in O’Keeffe *et al* (2014) [37].

### Culture media contaminants database

As it was not possible to fully remove culture media from specimens prior to proteomic analysis, a custom contaminates database was generated. This allowed for exclusion of protein identifications deriving from culture media (HDMMM) which contained porcine liver and baker’s yeast. Spectra obtained from LC-MS/MS of HDMMM were interrogated (MaxQuant and Perseus) against combined proteomic database of *Sus scrofa* and *Saccharomyces cerevisiae*, resulting in the identification of 2,135 proteins (min. 1 peptide). These proteins were added to MaxQuant contaminants database to generate a custom contaminants database (*n* = 2,380 contaminants).

### Data analysis of *D*. *pteronyssinus* proteomes

Spectra obtained from LC-MS/MS were interrogated (MaxQuant and Perseus) using either standard contaminant (HD samples) or custom contaminant (MB & SM samples) databases. Proteins were considered present when a minimum of two peptides (1 unique) for each parent protein was observed. Proteins meeting the following criteria were included in the analysis; (i) identified in two of the four non-fractionated whole protein extracts or (ii) identified in one chromatographic fraction.

### Qualitative assessment of mite group allergen localisation

A qualitative assessment of localisation of mite group allergens to MB or SM was conducted. An allergen was considered present in MB/SM proteome if it was; (i) absent from one dataset or (ii) found at higher LFQ intensity and ms/ms count.

## Results and discussion

### Phylogenomic assessment of *D*. *pteronyssinus* reveals closest relative to be *Euroglyphus maynei*

**Fig 1:**
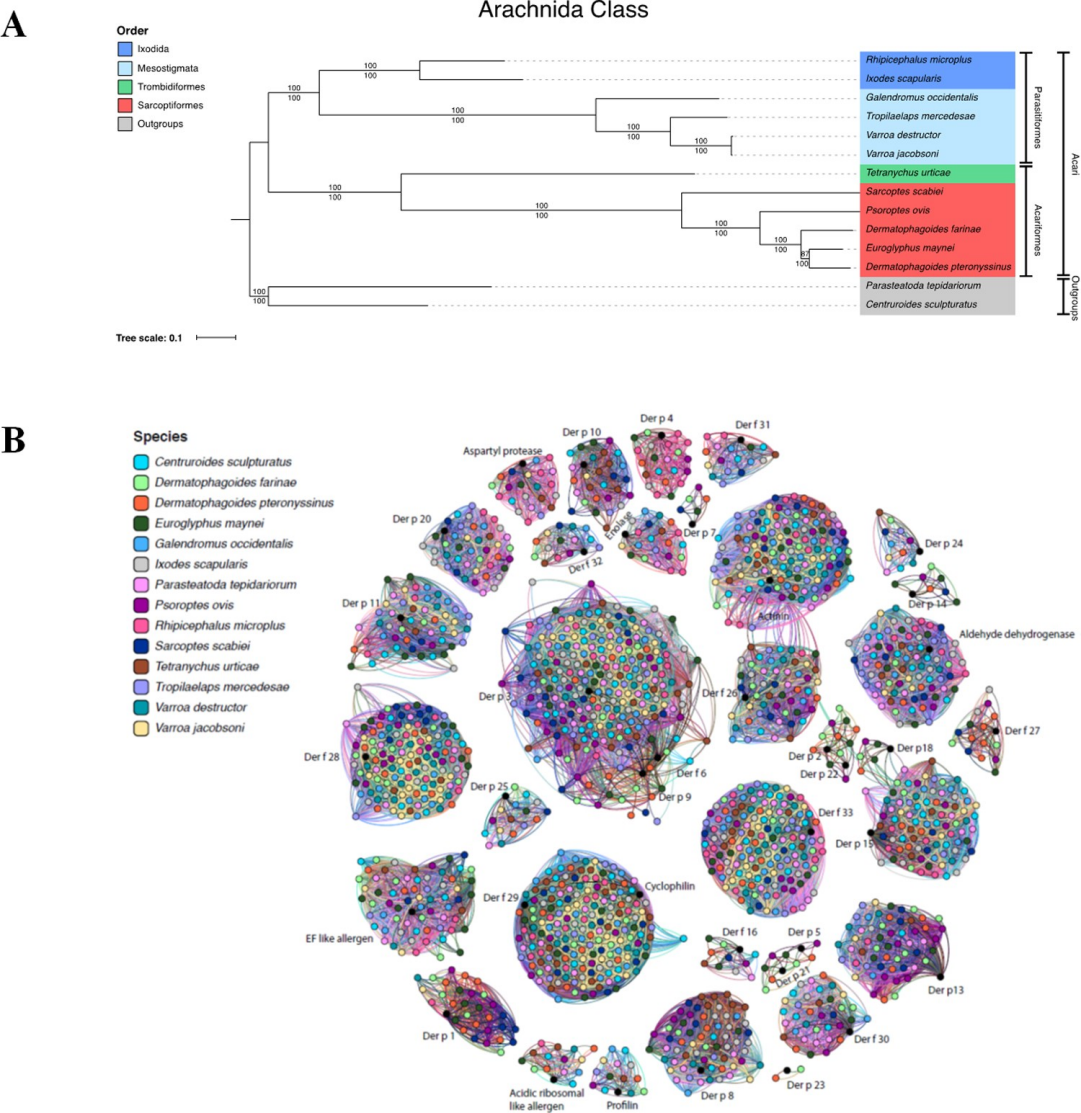
Arachnida phylogenomic species tree and network of mite group allergen orthologs in genomes of twelve Acari and two Arachinds. **A**. Phylogenomic species tree of 11 Acari species inferred using maximum-likelihood supermatrix (111 ortholog families, 77,878 aa aligned, LG+G+I+F model) and supertree (2,796 ortholog families) methods. Bootstrap support values are given for each branch, with values above branches corresponding to the supermatrix phylogeny and values below corresponding to the supertree phylogeny. Both supermatrix and supertree methods infer a strongly supported (87% and 100% bootstrap support respectively) sister group relationship between *D*. *pteronyssinus* and *E*. *maynei* to the exclusion of *D*. *farinae*. Therefore, in evolutionary terms *D*. *pteronyssinus* and *E*. *maynei* are more closely related to one another even though *D*. *pteronyssinus* and *D*. *farinae* belong to the same genus. **B**. Network map depicting abundance of mite group allergen homologs in each species. Homologous allergen group together forming an interconnected network distinct from non-homologous allergens i.e 2 & 22, groups 3, 6 & 9, groups 5 & 21, and groups 15 & 18.

Supermatrix and supertree phylogenomic methods were employed to infer the evolutionary relationships between the Acari species that have genome data available (S1 Table). Both supertree and supermatrix methodologies generated phylogenies with identical topologies and similarly high levels of bootstrap support (BP) for the monophyly of the Parasitiformes and Acariformes superorders (Fig 1A, 100% BP). Within the Parasitiformes superorder, Ixodida and Mesotigmata are monophyletic (Fig 1A, 100% BP). Within the Acariformes superorder, Trombidiformes and Sarcoptiformes are also found to be monophyletic (100% BP) although only a single representative of the Trombidiformes (*Tetranychus urticae*) is represented in our dataset (Fig 1A).

To date, phylogenetic studies of Acari (mites and ticks) have been restricted to multi-locus studies utilising a small number of genes, due to the absence of full genome sequences [38–40]. Our phylogeny infers a strongly supported (87% and 100% BP for supermatrix and supertree methods, respectively) sister group relationship between *D*. *pteronyssinus* and *Euroglyphus maynei* to the exclusion of *D*. *farinae*. This confirms previous studies which utilised only two and six genes but observed the same phylogenetic relationship between *D*. *pteronyssinus* and *E*. *maynei* [38, 41]. Therefore, in molecular evolutionary terms, *D*. *pteronyssinus* and *E*. *maynei* are more closely related to one another, even though *D*. *pteronyssinus* and *D*. *farinae* are currently classified within the same genus. Furthermore, the phylogeny confirms the paraphyly of the *Dermatophagoides* genus as previously reported [41].

### Comparison of *D*. *pteronyssinus airmid* predicted proteome with other *D*. *pteronyssinus* assemblies

*D*. *pteronyssinus airmid* genome assembly [27] completeness was assessed by proteo-genomics. Peppy software [26] facilitated the mapping of 615,150 spectra (28,001 non-redundant) to the predicted proteome and 402,998 (21,505 non-redundant) to the assembly. Peptides that spanned intron-exon junctions identified in the predicted proteome were not mapped to the assembly. Of the 21,505 peptides mapped to the genome assembly, 96.2% were also identified in predicted proteins (*n* = 20,683). Several peptides (*n* = 65) were located adjacent to predicted genes indicating that 65 gene models may need to be extended. The predicted proteome of *D*. *pteronyssinus airmid* [27] incorporates 8.3 million amino acids, this is significantly more than the Liu *et al*. (2018) and Randall *et al*. (2018) assemblies, which have 6.7 million and 5.9 million amino acids respectively. Moreover, despite having fewer predicted proteins than the other two available *D*. *pteronyssinus* proteomes (i.e. 12,530 versus 15,846 proteins [19] and 19,368 [18]), *D*. *pteronyssinus airmid* has on average longer protein coding genes (557aa vs. 425aa & 304aa respectively). These results indicate that differences in gene calling methodologies are most likely responsible for the differences in the number of protein coding genes.

Proteogenomic comparison of the predicted proteomes reveals the highest number of proteins were identified using the Waldron *et al* (2017) proteome (*n* = 4,581), followed by Randall *et al* (2018) with 4,416, then Liu *et al* (2018) with only 3,408 proteins identified. Therefore, unsurprisingly proteogenomic analysis of the available *D*. *pteronyssinus* genome assemblies against our protein samples which are derived from *D*. *pteronyssinus* airmid reveals the Waldron *et al* (2017) assembly and predicted proteome to be the most appropriate for our analyses, as higher numbers of protein identifications are uncovered relative to the other current assemblies [18, 19, 27].

### Mite group allergen orthologs in arachnidia

The presence of common MGA in the genomes of Arachnida species (S1 Table) was investigated, identifying multiple putative cross-reactive MGA orthologs in mite species. Most MGA had numerous orthologs distributed across all species (S1 Data) with presence closely linked to phylogeny. To help visualise the abundance of MGAs in the different Arachnida species a homology network was generated. Our results show that *D*. *farinae*, *D*. *pteronyssinus*, and *E*. *maynei* contained at least one MGA ortholog for all groups investigated, with the exception of Groups 23 & 24 in *E*. *maynei*. Group 7 and 14 allergens are only located in the Sarcoptiformes subset of Acariformes. Homologous allergens, Der p 5 and Der p 21, were present in the closely related Acariformes *D*. *pteronyssinus*, *D*. *farinae*, *E*. *maynei* and *Psoroptes ovis*, but absent from the other species (Fig 1B and S1 Data). Group 23 allergens are specific to *D*. *farinae* and *D*. *pteronyssinus*. More distantly related species from the Parasitiformes superorder were either missing orthologs of particular allergens such as Der p 4, 5 & 21 or contained RBH homologs only, Der p 1, 2, 22, 23 & 27 for example, (S1 Data). Serine proteases (groups 3, 6 & 9) appear to be expanded in some species, with a minimum of 10 homologs in *D*. *pteronyssinus* to a maximum of 28 in *ixodes scapularis* (Fig 1B and S2 Data).

### Annotation of *D*. *pteronyssinus airmid* predicted proteome

#### Predicted proteome annotations

**Fig 2:**
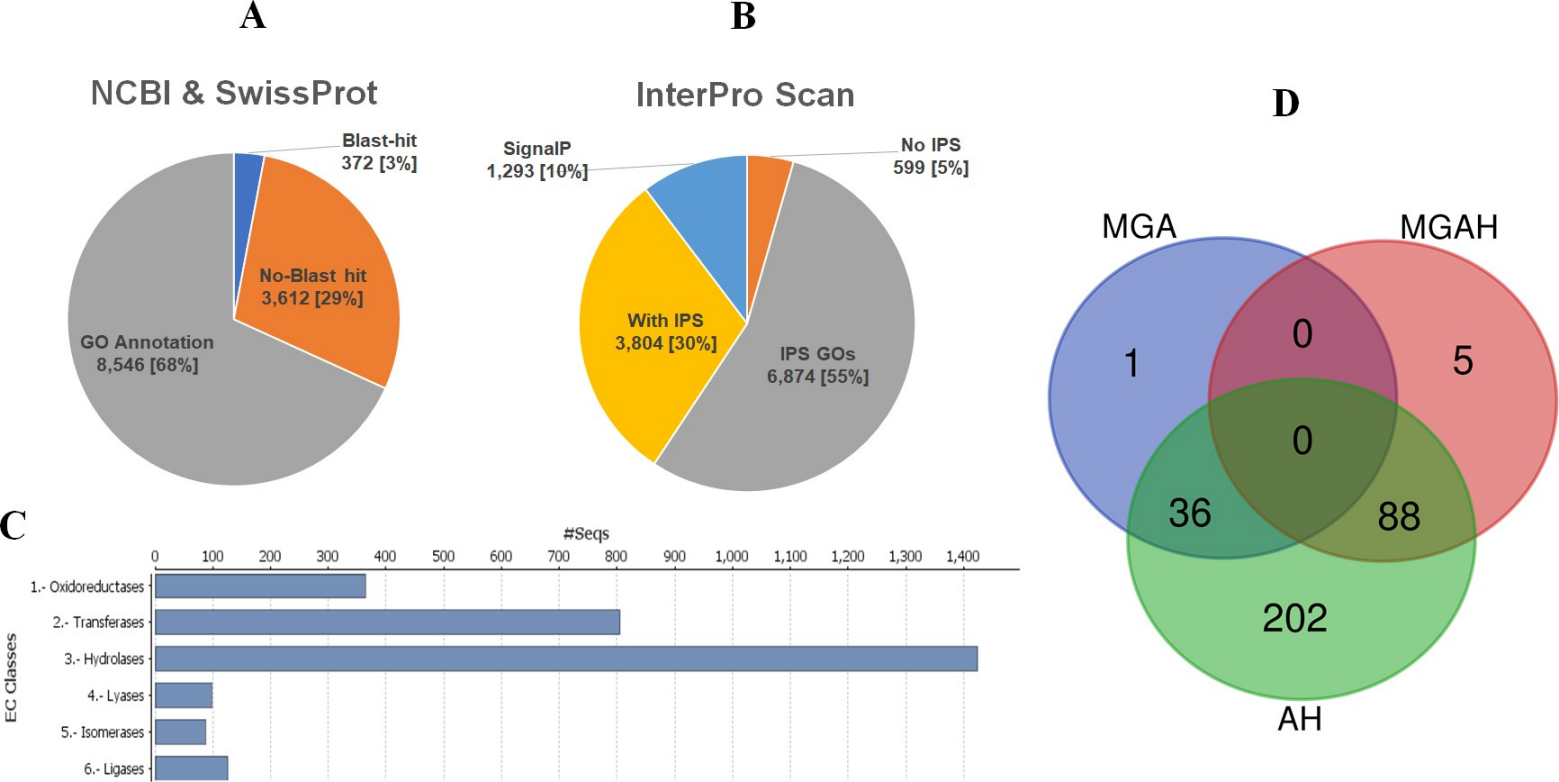
Annotation of *D*. *pteronyssinus airmid* predicted proteome. Annotation of *D*. *pteronyssinus airmid* proteins conducted using Blast2GO to search SwissProt, NCBI and InterPro databases for homology to known proteins and functional domains. **A**. GO annotations were assigned to 68.2% of the predicted proteome (*n* = 8,546). A small number of proteins had Blast hits with proteins in NCBInr or SwissProt databases but were not assigned a GO term (*n* = 372). The remaining proteins (*n* = 3,612) were not homologous with proteins in these databases. Most of the predicted proteins were annotated with more than one GO category (Biological Processes, Molecular function, Cellular component) with median number of assigned GO terms being 9 (Range: 0–164). **B.** InterPro scan feature of Blast2GO enabled assignment of InterPro IDs to 95.5% of predicted proteins/proteome? (*n* = 11,971). InterPro GO IDs were assigned to 55% (*n* = 6, 874) of the predicted proteome and predicted eukaryotic secretion peptides to 10.3% of the predicted proteome (*n* = 1,293). A further 3, 804 proteins were assigned InterPro Scan (IPS) IDs. Multi-database Blast2GO workflow enabled 96% of the predicted proteome to be assigned some form of annotation (*n* = 11,996). A small number of proteins had BLAST hits with proteins in NCBInr/SwissProt databases but were not assigned a GO term (*n* = 372). Several predicted proteins (*n* = 294) were assigned GO annotations but BLASTp hit alignments did not meet the required threshold of significance. The remaining proteins (*n* = 3,612) were not homologous with proteins in these databases **C**. Enzyme code classes assigned to *D*. *pteronyssinus airmid* predicted proteins (*n* = 2,689), representing 21.5% of *D*. *pteronyssinus airmid* predicted proteome. Hydrolyses (EC:3.0) formed the largest enzyme category (*n* = 1,244) and Isomerases (EC:5.0) the smallest (*n* = 87). **D.**Venn diagram depicting overlapping categorisation of allergenic and predicted allergenic proteins. *D*. *pteronyssinus airmid* predicted proteins were assigned into unambigous allergen goupings based upon BlastP homology to known allergens. We found full predicted proteins for all reported MGA (Groups 1–11, 13–16, 18, 20–33) and Seven non-chronalogical allergens (81.5–100% identiy). Subsequent blast hits were considered MGAH. Predicted proteins with potential cross-reactivity [32] with allergens from other species (Uniprot “allergenome”) were annotated as Allergen homolog (AH). Many Allergenic/potentially allergenic proteins were present in multiple allergen analyses, in total 332 allergenic/potentially allergenic proteins were identified.

Multi-database Blast2GO workflow enabled annotation of 96% of the predicted proteome (*n* = 11,996, S3 Data). Gene Ontology (GO) terms were assigned to 68.2% of proteins (*n* = 8,546, Fig 2A). InterPro Scan (IPS) assigned IPS annotations to 95.5% of predicted proteins (*n* = 11,971), 6, 874 with IPS GO terms and IPS IDs to a further 3,804 proteins (Fig 2B). SignalP4.0 identified eukaryotic secretion signals in 10.3% of predicted proteins (*n* = 1,293, Fig 2B). Enzyme codes (EC) were assigned to 21.5% of the predicted proteins (*n* = 2,689, Fig 2C) with hydrolases (EC:3.0) representing the largest enzyme category (*n* = 1,244). Putative peptidase activity was identified in 377 predicted proteins comprising almost 3% of the total predicted proteome. Peptidase EC (EC:3.4) were assigned to 275 peptidases, the remaining peptidases (*n* = 102) were identified by GO annotations. Enzymes have a propensity to cause allergy [42]. The potent peptidase activity of Der p 1 has been shown to disrupt numerous immune system processes [43] and it is thought lesser studied peptidases may have a similar effect [42]. Enzymes, particularly those with predicted secretion peptides, should be considered in the context of patient exposure as they are more likely to be excreted into house dust and therefore may augment the immune response.

#### *D*. *pteronyssinus* specific proteins

Predicted proteins without BLASTp alignments (*n* = 3,906) to proteins in NCBI/Swissprot were searched (E-value ≤1E^-05^) against closely related species (S1 Table), 2,054 had homologs in one or more species. The remaining 1,848 uncharacterised proteins (S4 Data) represented *D*. *pteronyssinus*-specific proteins [44]. Of these, 1,475 (S4 Data), were specific to *D*. *pteronyssinus airmid* strain as they were not found in the other *D*. *pteronyssinus* assemblies [18, 19]. These data suggest that 88.3% of identified proteins are core protein coding genes as they are found in all 3 *D*. *pteronyssinus* assemblies, with the remaining 11.7% being strain specific. Some uncharacterised *D*. *pteronyssinus airmid*-specific proteins may represent adaptations while others are a likely consequence of genetic drift occurring in isolated populations [45]. Bacterial pan-genomic studies estimate that strain-specific genes range from 5% to 35% per genome [46]. These strain-specific accessory genes are generally under relaxed mutational pressure, accumulating mutations more frequently than those of the core genome [47]. Further validation of the expression of these strain specific proteins is necessary to determine if they are functional proteins facilitating strain specific adaptations. It is worth highlighting that within the *D*. *pteronyssinus* representative proteome (discussed in more detail later) were 172 *D*. *pteronyssinus-*specific proteins, 23 of which had homologs in other *D*. *pteronyssinus* assemblies [18, 19], while 149 were only found in *D*. *pteronyssinus airmid*. The putative functions of these strain specific proteins are unknown, their role in strain specific adaptions may be discovered through further proteomic investigation.

#### LEA-like *D*. *pteronyssinus airmid* predicted proteins

HDM lose water readily through evaporation when the critical equilibrium humidity falls below optimum levels [48, 49]. Studies of biochemical mechanisms to resist desiccation have revealed late embryogenesis abundant proteins (LEAPs) play a key role in plant, insect and nematode desiccation survival [50–52]. Our analysis revealed 18 *D*. *pteronyssinus airmid* predicted proteins (S2 Table) to have significant homology with reported LEA proteins [30]. Gusev *et al* (2014) used a similar bioinformatic approach to identify 27 LEA-like proteins in the anhydrobiotic sleeping chromatid, *Polypedilum vanderplanki* [53]. This anhydrobiotic organism can tolerate extreme water loss of 97% by entering a state of suspended animation. The presence of similar proteins in *D*. *pteronyssinus* may explain the ability of mites in the protonymph developmental stage being entirely resistant to desiccation [49]. Although *D*. *pteronyssinus* can be killed by extended exposure to sub-critical equilibrium humidity, reduction of humidity in the home does not lead to a reduction in mite numbers or levels of allergen [54], as HDM return to a normal metabolic and reproductive state following short periods of optimal humidity. Furthermore, mattresses when occupied provide ample humidity to ensure survival of HDM in low humidity homes [14].

Expression of *D*. *pteronyssinus airmid* proteins exhibiting LEA-like proteins (*n* = 7) was validated by proteomics. All LEA-like proteins were found at low intensity (~LQF intensities of 1E+08) except for DERPT_G12026 and DERPT_G404 (LFQ Intensities > 7.8E+10). These two highly abundant LEA-like proteins are expressed under optimal non-desiccating laboratory growth conditions and were identified in both mite body and spent media. The ability of *D*. *pteronyssinus airmid* to utilise LEA-like proteins under normal laboratory conditions or in response to desiccation/cold may yield information that could be exploited for biocontrol strategies. The role of LEA-like proteins in relation to *D*. *pteronyssinus* is yet to be determined, however our data highlights them as potential players in desiccation resistance and hence as interesting biocontrol targets.

### Allergens and predicted allergens

We searched the *D*. *pteronyssinus airmid* proteome for the presence of 37 prevously reported mite allergens [7, 31]. MGA were identified for *D*. *pteronyssinus airmid*, we found full protein sequences (*n* = 37) for all reported MGA; Groups 1–11, 13–16, 18, 20–33 and seven non-chronological allergens (81.5–100% identity, Table 1). Subsequent BLAST hits (E-value ≤1E^-03^) were considered MGA homologs (*n* = 233, Table 1 and S5 Data). Der p 1-like cysteine proteases were represented by 31 homologs (20.9–63.7% identity), several were found in clusters of 2–3 adjacent protein coding genes (*n* = 13). MGA homologs with high sequence similarity to the query MGA (> 67% identity) met criteria for being considered isoallergens [55]. Isoallergens were identified for eight different MGAs (Table 1). Der p 28 has five isoalllergens (69.4–85.7% identity) and two were identified for Der p 29 (75.7–88.1% identity). One-third of MGA homologous proteins met the criteria for potential allergenicty [32] and were annotated as MGA Homologs (MGAH) (*n* = 93, S5 Data). In addition, many *D*. *pteronyssinus airmid* predicted proteins (*n* = 326, S6 Data) also exhibited significant similarity [32] to allergens from other species, suggesting they may be cross-species allergens. Most of these allergen homologs (AH) had multiple high scoring alignments with non *D*. *pteronyssinus* allergens (*n* = 991, Range:1–204, Median: 4). Significant overlap was seen between allergenic (MGA) and predicted allergenic (MGAH & AH) proteins with many being observed in more than one category, illustrated in Fig 2D. The structure and function of a protein has important implications for allergenicity, most allergenic proteins are limited to just 2% of protein families, with cross-reactivity linked to protein family rather than allergen source [6, 56, 57]. Most potential allergens highlighted in this study had predicted biochemical functions that placed them in well defined allergen families [6]. For example, predicted enolases DERPT_G12026 and DERPT_G4831 have high levels of sequence similarity (67–87%) with enolases from up to 19 phylogenetically distinct species (S6 Data). Enolase has long been recognised as a major cross-reacting allergen in plants, fungi, fish, and arthropods [58]. Moreover, the presence of at least one putative cross-reactive enolase ortholog [32] in all 12 Acari and two Arachnid outgroups (S2 Data) highlights the importance of this pan-allergen protein family. Several cyclophilins (*n* = 12) were annotated as putative cross-reactive proteins, of note Der f 29 like allergen (DERPT_G9923) exhibited sequence homology (52–83% identity) with cyclophilins from 12 different species (S6 Data). Cross-reactive cyclophilins from HDM, mouse, humans and fungi are well reported in the literature [59–61]. HDM and fungi are frequently co-present in HD [62] with A*lternaria* and *Aspergillus spp*. being the most common source of mould allergens [63]. *D*. *pteronyssinus airmid* predicted proteins exhibited homology to *Aspergillus fumigatus* (*n* = 19) and A*lternaria* alternata (*n* = 6) allergens. Homology between HDM and fungal proteins may play a role in reported fungal exacerbation of HDM-induced asthmatic symptoms [64, 65]. Excretion of putative allergenic proteins into HD *via* faecal particles would implicate a route of exposure and therefore has significant implications for allergy. Eukaryotic secretion peptides were predicted in 20.7% of allergenic/potentially allergenic proteins. Even if these putative allergens were unable to induce immune responces in their own right, co-presence with other immune modulators may be involved in bystander sensitization. Der p 1 accumulates in HD, levels exceeding 2 μg/g of dust are considered hazardous to the occupants [1]. In addition to being a potent activator of the immune system, Der p 1 has an adjuvant effect, enhancing IgE production against bystander molecules that may be present in HD [66]. Therefore, any protein accumulating in HD should be considered in the context of being a bystander allergen candidate.

**Table 1:** Mite group allergens (MGA), isoallergens and MGA homologs.

Group^A^ Allergen	Query Sequence ID^B^	Designated MGA Sequence ID^C^	Description^D^	Biochemical Function	% Identities	E-value	Isoallergens	No. Homologs^E^
1	P08176	DERPT_G1283	Der p 1	Cysteine protease 6	99.7	0		31
2	Q1H8P8	DERPT_G8792	Der p 2	Lipid binding	97.9	2.0E-104		6
3	P39675	DERPT_G8859	Der p 3	Trypsin 2	98.1	0		41
4	Q9Y197	DERPT_G360	Der p 4	a-Amylase 3	100	0	DERPT_G359	2
5	P14004	DERPT_G7260	Der p 5	Structural protein 2	99.2	1.1E-91		2
6	DEFA_160240	DERPT_G9187	Der f 6 like Allergen	Serine proteases	83.8	5.4E-140		39
7	P49273	DERPT_G8042	Der p 7	Unknown	99.5	8.3E-156		4
8	Q2YFE5	DERPT_G10534	Der p 8	Glutathione transferase 2	99.5	3.7E-166	DERPT_G10535	5
9	Q7Z163	DERPT_G7100	Der p 9	Serine protease	98.5	0		35
10	O18416	DERPT_G8047	Der p 10	Tropomyosin 5	99.3	0		5
11	Q6Y2F9	DERPT_G8381	Der p 11	Paramyosin 11	99.2	0		6
13	E0A8N8	DERPT_G8964	Der p 13	Fatty acid binding 2	100	3.3E-91		8
14	Q8N0N0	DERPT_G9820	Der p 14	Vitellogenin: egg yolk storage 6	99.6	0		3
15	Q4JK69	DERPT_G3350	Der p 15	Chitinase	99.3	0		13
16	A0A291KZA1	DERPT_G5615	Der f 16 like Allergen	Gelsolin: actin binding	99.8	0		2
18	Q4JK71	DERPT_G784	Der p 18	Chitinase	96.3	0		11
20	B2ZSY4	DERPT_G59	Der p 20	Arginine kinase	100	0	DERPT_G10997	3
21	Q2L7C5	DERPT_G7259	Der p 21	Structural protein 2	100	6.1E-98		2
22	A0A291KZA0	DERPT_G5267	Der f 22 like Allergen	MD-2–related lipid recognition	100	1.7E-106		7
23	L7N6F8	DERPT_G11207	Der p 23	Chitin-binding domain type 2	100	4.9E-65		14
24	A0A0K2GUJ4	DERPT_G5941	Der p 24 Allergen	Ubiquinol-cytochrome c reductase	100	3.1E-86		1
25	A0A291KYZ7	DERPT_G6990	Der f 25 like Allergen	Triosephosphate isomerase	100	0		2
26	A0A088SAG5	DERPT_G10519	Der f 26 like Allergen	Myosin light chain	92.4	7.6E-99		9
27	A0A291KZ97	DERPT_G8127	Der f 27 like Allergen	Serpin	82	0	DERPT_G8125 DERPT_G8126 DERPT_G8127	14
28	A0A291KZD8	DERPT_G6942	Der f 28 like Allergen	Heat shock protein 70	99.5	0	DERPT_G4999 DERPT_G585 DERPT_G7298 DERPT_G7750 DERPT_G9299	16
29	A1KXG2	DERPT_G9923	Der f 29 like Allergen	Cyclophilin	83.2	9.7E-111	DERPT_G1407 DERPT_G9923	14
30	Q962I7	DERPT_G8780	Der f 30 like Allergen Ferritin	Ferritin	100	5.7E-136	DERPT_G4895 DERPT_G9802	4
31	A0A088SAY1	DERPT_G5053	Der f 31 like Allergen	Cofilin	100	1.7E-108		1
32	A0A291KZC9	DERPT_G5605	Der f 32 like Allergen	Pyrophosphatase	100	3.0E-174		1
33	A0A2L0EBJ4	DERPT_G7263	Der f 33 like Allergen	a-tubulin	100	0	DERPT_G10901 DERPT_G8894	12
Non-Chronological Allergens	DEFA_097280	DERPT_G5569	Der f Acidic ribosomal like Allergen	Alpha Actin	81.6	1.3E-37		18
	L7UZ85	DERPT_G9110	Der f Actinin like Allergen	Aldehyde dehydrogenase	88.7	2.8E-141		11
	DEFA_095270	DERPT_G5156	Der f Aldehyde dehydrogenase like Allergen	Acidic Ribosomal Protein	88.8	0		3
	X4ZE83	DERPT_G12026	Der f Enolase like Allergen	Elongation Factor	93.8	0		15
	DEFA_072440	DERPT_G5247	Der f EF Elongation Factor like Allergen	Enolase	98.5	0		2
	DEFA_098190	DERPT_G10356	Der f Eukaryotic aspartyl protease like Allergen	Eukaryotic aspartyl proteas	86.2	0		1
	DEFA_029120	DERPT_G5894	Der f Profilin like allergen	Profilin	96.2	3.6E-93		1

Allergen Group^A^, Chronological and non-chronological allergens described for mites. Query Sequence ID^B^, UniProt Accession or *D*. *farinae* Accession (Chan *et al*., 2015). Sequence ID^C^, *D*. *pteronyssinus airmid* protein sequence ID. Description^D^, mite group allergen name.

*D*. *pteronyssinus airmid* protein sequences designated as MGA inclusive of (Der p1-11, 13–16, 18, 20–33, *n* = 30) and non-chronalogical allergens (*n* = 7) based on BLASTp identity to MGA sequences available in UniProt for *D*. *pteronyssinus* and *D*. *farinae* (where *D*. *pteronyssinus* sequences were unavailable). Where more than one BLAST result was returned, the highest scoring hit was chosen to represent the MGA. Subsequent BLAST hits were annotated as MGAH. Several MGAH were homologous with more than one query sequence, i.e multiple predicted proteins were homologous with Der p3, Der p6 and Der p9. MGAH exhibiting >67% identity with query allergen were annotated as Isoallergen

### Proteomic characterisation of *D*. *pteronyssinus airmid*

#### Representative proteome

**Fig 3:**
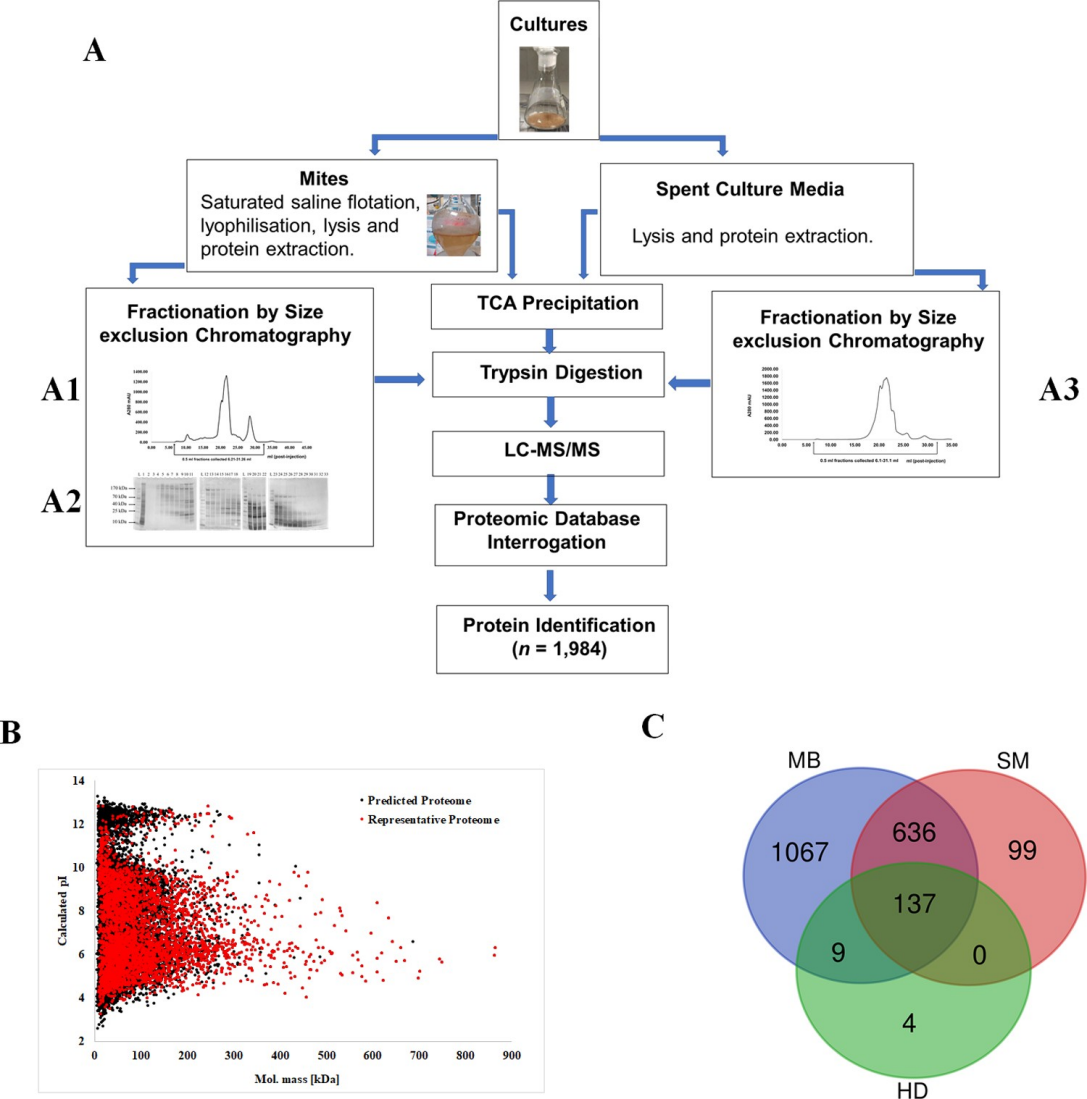
Proteomic characterisation of *D*. *pteronyssinus airmid*. **A.** Proteomic Strategy for characterisation of *D*. *pteronyssinus airmid*. Flow diagram depicting proteomic strategy for characterisation of *D*. *pteronyssinus airmid* excretome and mite body proteome utilising whole sample analysis and sample fractionation analysis. **A1**. Gel filtration chromatography of *D*. *pteronyssinus airmid* whole body homogenate. Whole body homogenate protein extract (4.5 mg; 500 μl injection) separated by size exclusion chromatography (Superdex 200 10/300 GL; 0.4 ml/min flow rate). 0.5 ml fractions collected between 6.21 and 32.26 ml post-injection. **A2.** SDS-PAGE analysis of *D*. *pteronyssinus airmid* whole body homogenate fractions. SDS-PAGE (4–20%) with silver staining of whole body homogenate protein extract (Lane 1; 10 μg) from *D*. *pteronyssinus airmid* and fractions from gel filtration (6.2–22.2 ml post-injection) of same (Lanes 2–13, 17 μl loaded). SDS-PAGE visualisation of fractions (0.5ml) depicting fractionation of complex protein mixture into reduced complexity extracts containing proteins of approximately similar sizes. Fractions, 7.2–32.2 ml, post injection (*n =* 50) were pooled to give 1–1.5 ml aliquots (*n* = 21),100 μl was processed for LC-MS/MS. **A3.** Gel filtration chromatography of *D*. *pteronyssinus airmid* spent growth medium (SM). Protein extract of *s*pent growth media (0.2 mg; 500 μl injection) separated by size exclusion chromatography (Superdex 200 10/300 GL; 0.4 ml/min flow rate). 0.5 ml fractions collected between 6.1 and 32.1 ml post-injection for SDS-PAGE analysis. Proteins in gel filtration fractions were acidic and therefore unsuitable for analysis by SDS-PAGE. Therefore, fractions were pooled and analysed according the methods used for mite body extract fractions. Fractions, 7.1–32.1 ml, post injection (*n =* 50) were pooled to give 1–1.5 ml aliquots (*n* = 21), 100 μl was processed for LC-MS/MS. **B.** Alignment of theoretical molecular mass and pI from *D*. *pteronyssinus airmid* PP *(n* = 12, 530) and representative proteome (*n* = 3,931) Software: http://www.juirgel.de Hiller et al., (2006). Proteins > 900 kDa excluded for graphing purposes (DERPT_G11449, DERPT_G9606, DERPT_G4775, DERPT_G8007,3088 kDa, 2021 kDa, 1122 kDa & 1001 kDa respectively). Predicted proteins with strong proteomic evidence for expression (2+ medium confidence peptides) accounted for 31.4% of the predicted proteome (*n* = 3, 931). Calculated molecular mass and pI (Range: 5.25–3086.2 kDa, pI 3.26–12.85) of representative proteome was approximate to that of the predicted proteome (Range: 3.94–3086.2 kDa, pI 2.63–13.27). Median protein size in the *D*. *pteronyssinus airmid* PP was 54.25 kDa. The largest predicted protein coding gene was DERPT_G11449 at 79,548 bp and encoded a paladin (Titin) protein homolog (26,516 a.a, 3086.2 kDa, pI 4.93). The smallest predicted protein was DERPT_G12515 (36 a.a, 3.94 kDa, pI 6.04) of unknown function, encoded by a 106 bp gene. The median protein pI was calculated to be 7.42, the protein with lowest predicted pI of 2.63 was DERPT_G12367 (50 a.a, 5.45 kDa), DERPT_G11425 had the highest predicted pI at 13.27 (67 a.a, 6.94 kDa). **C.** Venn Diagram depicting overlap between Mite Body (MB), Spent Culture Medium (SM) and House Dust (HD) Proteomes, totalling 1,952 proteins identified.

Analysis of LC-MS/MS spectra obtained from proteomic analysis of *D*. *pteronyssinus airmid* resulted in the high confidence identification of 3,931 proteins (S7 Data). This representative proteome comprised 31.4% of the predicted proteome of *D*. *pteronyssinus airmid* with experimental evidence for expression. Protein molecular weight and pI were widely distributed in the representative proteome (Range: 5.25–3086.2 kDa, pI 3.26–12.85) and similar to that of the predicted proteome (Range: 3.94–3086.2 kDa, pI 2.63–13.27), confirming the protein extraction methods were optimal for the characterisation of *D*. *pteronyssinus airmid* proteome (Fig 3A). Establishing a representative proteome, that reflects the methodological limitations of protein extraction and identification is essential for subsequent enrichment analyses. This background proteome is defined by Bessarabova *et al* (2012) as “the complete set of proteins known to be expressed in an organ/tissue/body liquid/cell line of sample origin” [67]. To date, proteomic investigation of *D*. *pteronyssinus* has extended to a few discrete studies [68–71]. Laboratory HDM populations have been shown to have different reproduction rates to wild-type strains [72] and isolated populations to give rise to geographical allergen variants [7]. Experimental examination of uncharacterised *D*. *pteronyssinus airmid-*specific proteins, particularly those with evidence for expression, may provide useful insights into the genes evolving within isolated populations. Our representative proteome may be expanded by employing alternative protein extraction methods, use of trypsin alternative or multi-protease digestion and depletion of high abundance proteins [73–75].

#### Wildtype proteome of *D*. *pteronyssinus*

Extending the relevance of *D*. *pteronyssinus* proteomics to the environment is essential, as very little is known about which HDM components are present in inhalable air [10]. Proteomic analysis of 21 Der p 1 positive HD samples revealed the presence of 150 *D*. *pteronyssinus* proteins (S8 Data), the ten most abundant are given in Table 2, with sequence coverage ranging from 9.6–73.3%. Here, it can be seen that allergens Der p 1, 2, 5, 14 and Der f 6 like allergen are amongst the most abundant *D*. *pteronyssinus* proteins in HD. Der p 1 and Der p 2 are considered major allergens while Der p 5, 6 and 14 are mid-tier IgE binders, all were amongst the ten most abundant proteins in HD [7]. Five non-allergenic proteins were also highly abundant, suggesting that sensitisation and IgE binding is a result of the unique properties of the protein rather than its abundance in house dust. Allergenic/potentially allergenic molecules accounted for almost 51% of all proteins identified in HD (*n* = 76). The predicted functions of many of these proteins place them into well-established allergen families, therefore their presence in HD and sequence similarity to known allergens make them interesting targets for future studies seeking to identify new allergens. Comparative analysis between allergenic and non-allergenic components of HD may reveal epitopes or structural characteristics common to inhalant allergens [10]. Our data illustrates the utility of high sensitivity protein MS as a novel way to identify HDM products in the wild-type environment and has significant implications for the development of immunotherapies that mimic natural exposure. Previously, researchers have examined numerous commercially available diagnostic and therapeutic HDM extracts, finding many were lacking important allergens and some had several fold variations in Der p 1 to Der p 2 ratios [76]. For example, the 2017 international consensus (ICON) report on the clinical consequences of mite hypersensitivity states that the “development of objective methods to assess allergen exposure and environmental control outcomes” are unmet and in need in mite allergy research [1]. Furthermore, the European medicines agencies guideline on the clinical development of products for specific immunotherapy for the treatment of allergic diseases, states that for seasonal allergies “it is mandatory to document the exposure to the relevant allergens” and “it is recommended to document the exposure level for the individual patient especially for the evaluation periods to evaluate the variation of indoor allergens” [77]. Generating diagnostic and therapeutic HDM extracts with allergen content and ratios that mimic natural exposure is of great importance. Characterisation of the factors affecting allergen repertoire and accumulation in different home microhabitats may give rise to much needed strategies for reducing allergen exposure for sensitised individuals [78].

**Table 2:** Top 10 most abundant *D*. *pteronyssinus* proteins identified in house dust protein extracts.

Sequence IDs^A^	Description^B^	N: Samples^C^	N: Unique peptides^D^	Sequence coverage [%]	Mol. Mass [kDa]	N: MS/MS count^E^	LFQ Intensity^F^
DERPT_G8792	Der p 2 Allergen; Proposed Mon-Allergen, Allergen Homolog (Der f2, Eur m2, Der s2)	21	7	56.2	15.9	178	4.3E+10
DERPT_G1283	Der p 1 Allergen, Proposed Sten-Allergen, Allergen Homolog (Der f1, Der m1, Pso o1)	21	11	18.8	96.0	129	1.9E+10
DERPT_G212	Polyubiquitin	21	9	18	287.2	133	1.5E+10
DERPT_G10213	Glutamate receptor kainate 3	17	12	17.7	99.7	97	5.1E+09
DERPT_G9820	Der p14 Allergen; Proposed Sten-Allergen, Allergen Homolog (Sar s14, Eur m14, Der f14)	17	51	36.2	191.4	212	4.9E+09
DERPT_G7260	Der p5 Allergen; Allergen Homolog (Der f5)	17	8	21.5	36.2	70	4.5E+09
DERPT_G4157	Der p36 Allergen	20	7	64.7	24.5	73	3.4E+09
DERPT_G114	Proposed Sten-Allergen, Allergen Homolog (ole e5, Sola 1 SOD)	13	7	74.3	15.7	60	3.4E+09
DERPT_G9187	Der f6 like allergen; Allergen Homolog (Blo t6)	18	11	9.6	228.1	122	3.2E+09
DERPT_G9697	Sucrase- intestinal, alpha-1,4-glucosidase activity	18	16	16.6	202.1	119	2.8E+09

Sequence ID^A^, *D*. *pteronyssinus airmid* protein sequence ID. Description^B^, annotations assigned by Blast2GO. N: Samples^C^, number of extracted dust samples in which specified protein was identified. N: Unique peptides^D^, number of unique (not present in any other protein sequence in the predicted proteome) peptides identified by LC-MS/MS for specified protein. N: MS/MS count^E^, sum of peptides selected for ms/ms analysis. LFQ Intensity^F^, label free quantification intensity. (Software: Maxquant version 1.6.2.10, Perseus version 1.6.2.2)

#### Proteome of laboratory-reared *D*. *pteronyssinus airmid*

*D*. *pteronyssinus airmid* whole protein lysates (WPL) were analysed directly (shotgun) and subjected to fractionation by size exclusion chromatography resulting in identification of 1,948 MB and SM proteins by high sensitivity protein mass spectrometry. Protein identification was confirmed by detection of at least two peptides per protein, and percentage sequence coverage ranged from 0.2 to 99.4% across the entire protein dataset. Gel filtration fractionation of MB extracts and SM (Fig 3A1–3A3) led to the unique identification of 248 and 105 proteins (18% of total identified proteins) from chromatographic fractions. Of the proteins identified, 1,076 proteins (58.2%) were uniquely found in MB extracts (S9 Data), while 99 proteins (0.8%) were solely identified as secreted proteins (S10 Data). Faecal rich SM was obtained by sieving to remove large mites, therefore smaller mites remained. In a previous proteomic study of *D*. *pteronyssinus* faeces, it was suggested that the method of faeces sample collection is beneficial over the sieving technique, which unavoidably contains mite bodies and growth media [69]. While culture media-derived proteins (contaminants) will also be detected in proteomic analysis, it is possible to differentiate true dust mite proteins from contaminants by use of a culture media contaminants database as demonstrated in our study. Several functional terms assigned to proteins were differentially represented in MB (S9A–S9C Data) and SM (S10A–S10C Data) proteomes compared to the RP.

Several GO terms were differentially represented (Over-represented: *n* = 161, Under-represented: *n* = 6, S9A Data), of note, GO Cellular Component terms cytosol (Fisher’s *P* = 5.3E-49, GO:0005829) and mitochondrion (Fisher’s *P* = 8.5E-33, GO:0005739) were the most highly over-represented terms. This finding supports our proteomic strategy, showing significant enrichment of GO terms associated with intracellular activities. EC were highly represented in the MB proteome, assigned to 44.6% of proteins identified (*n* = 824). Only two enzyme names were found to be differentially represented, acting on peptide bonds (Fisher’s *P* = 1.28E-05) was over-represented and transferring phosphorous-containing groups (Fisher’s *P* = 1.03E-04) underrepresented (S9B Data). NAD(P)-binding domain superfamily (IPR036291) was the most highly over-represented IPS ID (Fishers *P* = 1.63E-12) of 25, with mobidb-lite IPS ID (Fishers *P* = 5.58E-17) for Intrinsic disorder protein sequences, representing the most significantly underrepresented of 30 IPS IDs (S9C Data).

All MGA apart from Der p 28 were found in the MB, six were among the most abundant proteins identified (S3 Table). In the absence of Der p 28, nine Der p 28 homologs were identified including four isoallergens (Table 1 and S9 Data). MGA were also found to be amongst the most abundant proteins in the SM, including Der p 2 and Der p 14 (S4 Table). Allergenic/potentially allergenic molecules were highly represented in both MB and SM proteomes accounting for 11.4% and 18.5% of all proteins identified. Homologs of Der p 1 (*n* = 17), Der p 2 (*n* = 2) and Der p 23 (*n* = 1) were identified in the SM. Given that allergens Der p 1and Der p 2) account for up to 60% of IgE reactivity in HDM sensitised individuals [5], and Der p 23 sensitivity is seen in 79% of HDM allergic patients [79] examination of cross-reactivity between these MGA and excreted homologs is warranted.

MB and SM proteomes were abundant in enzymes, which accounted for 44.6% and 52.4% of all proteins identified respectively (S9 and S10 Datas), some of these enzymes may be involved in digestion. HDM have long been associated with feeding on shed skin present in HD. While they have been observed to eat skin, the poor nutritional value of keratin makes it unlikely to be a primary food source. Rather, HDM are trophic generalists, they feed on organic debris associated with their proximity to humans [16]. In the laboratory, *D*. *pteronyssinus* have been grown on diverse culture media including various combinations of wheat bran, wheat flour, dog food, rodent chow, ground porcine liver, dried egg powder, defatted skin scales, and fish food. Most research groups use dried yeast to supplement diets and improve mite population growth [72, 80–83]. HDM have been observed to feed on bacteria and fungi in laboratory experiments [84, 85]. Whether HDM consume bacteria or fungi in a wildtype setting as a nutrient source needs further experimental evidence. Expression and excretion of bacterial and fungal degrading enzymes may indicate a role in digestion [85].

Numerous glycoside hydrolases (EC:3.2.1) were identified in the predicted proteome (*n* = 57) and proteomic datasets (RP, MB & SM) summarised in Table 3. Each glycoside hydrolase enzyme sub-family were represented by at least one proteomic identification. Two predicted lysozymes (EC:3.2.1.17) and one 14.5 kDa bacteriolytic enzyme, Der p38 (DERPT_G10989) were identified with evidence for high expression, these enzymes may be responsible for bacteriolytic activity in HDM extracts [86]. Excreted proteins with predicted activities against major components of fungal cell walls were identified and include; chitinases (*n* = 5), four glycoside hydrolase family 16 members with putative β-1,3 glucanase activity, β-mannosidase (*n* = 1), α-mannosidases (*n* = 4), chitosanase (*n* = 1) and α-N-Acetyl hexosaminidaseine (*n* = 2). Carbohydrate metabolism GO terms were among the 318 over-represented GO terms (Fisher’s exact test < 0.05 FDR) in the excretome and included carbon utilization, hydrolase activity hydrolyzing O-glycosyl compounds, chitin metabolic process, chitin binding and starch metabolic process (S10A Data). Of the putative enzymes identified in the SM proteome (*n* = 457), enrichment analysis showed 35 to be over-represented, many of which related to carbohydrate digestion; Alpha-glucosidase, Chitinase and Alpha-mannosidase (S10B Data). The presence of a predicted secretion signal was a strong indicator of excretion as 22.5% of all proteins identified in the SM proteome contained predicted secretion signals. Moreover, secretion signal peptides were the most highly over-represented IPS ID (Fisher’s *P* = 1.08E-26) of 61 (S10C Data).

**Table 3:** Carbohydrate degrading enzymes.

	Proteomic Evidence				
Carbohydrate active enzyme	EC No/ InterPro ID	PP^A^	RP^B^	MB^C^	SM^D^
Glycosidases	3.2	63	44	32	30
Glycosyl hydrolases	3.2.1	57	42	30	29
a-amylase	3.2.1.1	2	1	1	1
Chitinase	3.2.1.14	10	8	3	5
Lysozyme	3.2.1.17	3	1	2	1
Alpha-Glucosidase	3.2.1.20	4	4	4	4
Alpha-Galactosidase	3.2.1.22	3	2	2	2
Beta-Galactosidase	3.2.1.23	3	2	2	2
Alpha-Mannosidase	3.2.1.24	11	8	5	5
Beta-Mannosidase	3.2.1.25	1	1	1	1
Trehalase	3.2.1.28	2	2	0	0
Beta-Glucuronidase	3.2.1.31	1	1	1	1
amylo-alpha-1,6-glucosidase	3.2.1.33	2	1	1	0
Hyaluronoglucosaminidase	3.2.1.35	2	0	1	0
Glucosylceramidase	3.2.1.45	2	2	2	2
Alpha-L-Fucosidase	3.2.1.51	3	2	2	2
Alpha-*N*-Acetyl hexosaminidase	3.2.1.52	5	5	2	2
mannosyl-glycoprotein endo-β-N-acetylglucosaminidase	3.2.1.96	1	1	0	0
Chitosanase	3.2.1.132	2	1	1	1
Glysoside Hydrolase family 16	IPR000757	6	4	3	14
Glyco Hydrolase family 18 (W/O EC)^F^	IPR001223	9	8	4	4

PP^A^, Predicted Proteome. RP^B^, set of all proteins identified in the Representative proteome. MB^C^, set of all proteins identified in the mite body. SM^D^, set of all proteins identified in the ExcExcretome. HD^E^, set of all *D*. *pteronyssinus* proteins identified in house dust. Glyco Hydrolase family 18 (W/O EC)^F^, proteins annotated as Glyco Hydrolase family 18 but lacking enzyme code annotation.

The expression of the numerous carbohydrate active enzymes listed above provides compelling evidence to support observations of *D*. *pteronyssinus* feeding on fungi and bacteria and demonstrate that they possess the necessary enzymes to utilise bacteria and fungi as a nutrient source [85]. Feeding on bacteria or fungi within wild-type microhabitats may alter allergen repertoire between homes, as diet has been demonstrated to alter allergen production in laboratory HDM cultures [87]. This new insight compounds the necessity for characterising factors affecting HDM allergen production within the home.

The process of chitin synthesis and remodelling is an integral part of the growth and development of all arthropods. Chitin remodelling enzymes include chitinase, β-N-Acetylhexosaminidase and the highly conserved chitin synthase, a key enzyme in the insect biosynthetic pathway [88]. Proteomic profiling of *D*. *pteronyssinus airmid* facilitated identification of eight predicted chitinases (EC:3.2.1.14, five β-N-Acetylhexosaminidases (EC:3.21.52) and two chitin synthases (IPR004835) (Table 3) putatively involved in chitin remodelling. These enzymes represent important biocontrol targets, as chitin is absent from vertebrates, dysregulation of these enzymes could provide a much needed method of curtailing HDM populations in the homes of sensitised individuals [12, 88].

### Mite group allergen localisation

All proteins are synthesised in the MB prior to excretion, however excreted proteins are likely to accumulate in growth medium and HD. Data regarding localisation of MGA are limited [7]. Localisation is often linked to the degree of protein allergenicity, identifying sites of MGA accumulation may reveal trends of exposure that can be applied to assessing new allergens. In our analysis, the majority of MGA were detected in MB and SM proteomes, the relative amounts in each proteome was used to infer localisation. Proteomic assessment of localisation showed sero-dominant allergens Der p 1, Der p 2 and Der p 23 to accumulate in SM (Table 4), with Der p 1 and Der p 2 being the two most abundant proteins (S4 Table). Der p 23 has previously been reported to be found only in low quantities in SM relative to Der p 2 [79], we observed the same trend, more Der p 2 ms spectra (*n* = 486) were detected than for Der p23 (*n* = 90). Allergens Der p 3, Der f 6 like allergen, Der p 9, Der p 15 and Der p 28 were also found to accumulate in the SM (Table 4). Localisation of Der p 3 to SM is consistent with previous observations for Der f 3 [89], the serine peptidases Der p 3, Der f 6 like allergen and Der p 9 were all among the top 10 most abundant SM proteins (S4 Table). Despite a different methodological approach, another study also found Der p 1, Der p 2, Der p 6 and Der p 15 to be major proteins in *D*. *pteronyssinus* faeces and SM [69].

**Table 4:** Proteomic evaluation of mite group allergen localization in *D*. *pteronyssinus airmid*.

MGA^A^	Sequence ID_B_	N: Mol. mass [kDa]^C^	N: Sequence coverage [%]^D^	N: Score^E^	N: MS/MS count^F^	N: LFQ Intensity^G^	Localisation^H^
1	DERPT_G1283	96.0	41.3	323.31	1511	4.9E+11	**SM**
			32.2	323.31	966	2.7E+11	
2	DERPT_G8792	15.9	78.8	323.31	486	5.2E+11	**SM**
			76.7	323.31	419	6.7E+11	
3	DERPT_G8859	28.1	67.4	323.31	510	4.4E+11	**SM**
			66.7	323.31	250	5.1E+10	
4	DERPT_G360	60.3	75.5	323.31	370	1.5E+11	**MB**
			76.9	323.31	410	1.8E+11	
5	DERPT_G7260	36.2	25.5	323.31	188	2.7E+10	**MB**
6	DERPT_G9187	228.1	14.4	323.31	468	2.6E+11	**SM**
			17.9	323.31	192	2.4E+10	
7	DERPT_G8042	28.5	48.6	256.04	105	1.7E+10	**MB**
			53.7	323.31	273	6E+10	
8	DERPT_G10534	25.6	57.1	248.79	65	5.3E+09	**MB**
			97.3	323.31	165	3.8E+10	
9	DERPT_G7100	29.4	87.2	323.31	440	2.9E+11	**SM**
			85.3	323.31	162	2.2E+10	
10	DERPT_G8047	33.0	77.1	323.31	171	3.9E+09	**MB**
			81.7	323.31	923	5.4E+11	
11	DERPT_G8381	184.0	48.9	323.31	1822	4.7E+11	**MB**
13	DERPT_G8964	15.0	71.8	222.22	66	4E+09	**MB**
			90.8	323.31	387	2E+11	
14	DERPT_G9820	191.4	78.5	323.31	1388	1.6E+11	**MB**
			88.8	323.31	4729	2.2E+12	
15	DERPT_G3350	183.4	27.1	323.31	706	3E+11	**SM**
			26.3	323.31	398	1.1E+11	
16	DERPT_G5615	55.4	62.1	323.31	127	6.1E+09	**MB**
			92.1	323.31	718	1.5E+11	
18	DERPT_G784	63.1	48	323.31	267	4.7E+10	**SM**
			45.3	323.31	218	4.6E+10	
20	DERPT_G59	43.8	72.4	323.31	196	2.6E+10	**MB**
			85.1	323.31	727	3.7E+11	
21	DERPT_G7259	16.5	14.3	10.742	1	1.2E+07	**MB**
			70.7	138.58	117	2.3E+10	
22	DERPT_G5267	15.6	51	135.52	58	1.9E+10	**MB**
			65	135.32	62	2.1E+10	
23	DERPT_G11207	10.3	44.4	323.31	90	6.6E+10	**SM**
			34.4	32.386	26	3.6E+09	
24	DERPT_G5941	16.6	49.3	36.632	37	1.4E+09	**MB**
25	DERPT_G6990	30.0	76.4	323.31	157	2.4E+10	**MB**
			80	323.31	293	1E+11	
26	DERPT_G10519	133.7	10.8	138.1	134	3.1E+10	**MB**
27	DERPT_G8127	43.3	37.8	247.65	19	3.3E+09	**MB**
			37.8	189.99	54	2E+10	
28	DERPT_G6942	72.5	23	33.474	22	2E+09	**SM**
29	DERPT_G9923	40.8	19.7	172.32	66	2.2E+10	**MB**
			27.7	155.96	122	4.8E+10	
30	DERPT_G8780	20.8	86.1	323.31	153	3E+10	**MB**
			99.4	323.31	572	6E+11	
31	DERPT_G5053	16.8	77	251.82	57	2.1E+09	**MB**
			89.2	275.91	154	3.9E+10	
32	DERPT_G5605	166.9	33.4	323.31	237	2E+10	**MB**
			37.3	323.31	286	5.2E+10	
33	DERPT_G7263	51.4	38.3	87.829	21	7.6E+08	**MB**
Der f acidic ribosomal like allergen	DERPT_G5569	11.5	45.1	49.195	8	1.7E+08	**MB**
			45.1	71.984	30	3E+09	
Der f Actinin like allergen	DERPT_G9110	149.6	20	219.08	45	3.8E+08	**MB**
			62.1	323.31	841	1.9E+11	
Der f aldehyde dehydrogenase like allergen	DERPT_G5156	54.1	94.1	323.31	303	2.7E+10	**MB**
			95.9	323.31	840	4.3E+11	
Der f EF elongation factor like Allergen	DERPT_G5247	94.7	70.1	323.31	297	2.6E+10	**MB**
			78.2	323.31	765	2.4E+11	
Der f enolase like Allergen	DERPT_G12026	47.4	78.8	323.31	261	4E+10	**MB**
			84.3	323.31	597	2.4E+11	
Der f eukaryotic aspartyl protease like allergen	DERPT_G10356	42.9	45.6	172.3	38	1.2E+09	**MB**
			62.2	166.75	137	1.8E+10	
Der f profilin like allergen	DERPT_G5894	14.3	73.8	111.29	30	2.4E+09	**MB**
			86.2	99.86	67	1.2E+10	

Highlighted in in gray: Mite Body(MB) proteome Data. In white: Excretome proteome data (SM). MGAA, Mite Group Allergen. Sequence IDB, D. pteronyssinus airmid protein sequence ID. DescriptionB, annotations assigned by Blast2GO. N: Mol. mass [kDa]C, calculated Molecular mass of protein in kDa. N: Sequence coverage [%]D, percentage of total protein sequence for which peptides were identified. N: ScoreE, protein score, cumulative score of individual peptide mass spectra’s. N: MS/MS countF, sum of peptides selected for ms/ms analysis. LFQ IntensityG, label free quantification intensity. (Software: Maxquant version 1.6.2.10, Perseus version 1.6.2.2). LocalisationH, qualitative assessment of allergen localisation to MB or SM based upon MS/MS count and LFQ intensity.

Analysis of localisation must not be restricted to laboratory-based studies and should include environmental reference samples where possible as demonstrated for allergens Der p 5 and Der p 21. Initial proteomic assessment found Der p 5 and Der p 21 were not excreted under laboratory conditions as they were absent from SM (S10 Data). However, proteomic analysis of HD revealed Der p 5 to be among the top 10 most abundant proteins (Table 2) and Der p 21 the 63^rd^ most abundant, both were identified in at least 16 of the Der p 1 positive dust samples (S8 Data). The location of Der p 5 and Der p 21 in laboratory cultures, present in MB and absent from SM, would indicate they are not excreted, however, their presence in HD shows that there must be other factors that contribute to accumulation of non-excreted allergens in the home. These allergens may accumulate in HD as the mite bodies begin to degrade. The accumulation of dead mites in laboratory cultures is avoided by regular sub-culturing of mites. Cross-referencing SM with MB associated proteins as demonstrated in Table 4, can infer localisation and can allow for novel insights into modes of accumulation when compared to wild-type dust samples. Our work has demonstrated the utility of high sensitivity mass spectrometry in characterising the complex proteomes of *D*. *pteronyssinus*. Laboratory cultures show parallels in protein expression with wild-type samples, as most proteins were identified in two or more of the proteomes (Fig 3C) with only four proteins uniquely identified in HD. Additional research is required to characterise the various microhabitats of HDM and factors affecting allergen presence in the home.

## Conclusion

Here we performed a comprehensive bioinformatic and proteomic examination of *D*. *pteronyssinus* airmid describing the expression of 4,002 proteins (S11 Data) and identified 332 potential allergens. High sensitivity mass spectrometry allowed for the description of novel *D*. *pteronyssinus* components in HD and facilitated qualitative assessment of MGA localisation. This research has expanded the knowledge of proteins utilised by *D*. *pteronyssinus* for key physiological processes and will form the basis for further research into biocontrol strategies for the medically important HDM.

## Supporting information

S1 Table Genome assemblies utilised for phylogenetic analysis. (DOCX)

S2 Table LEA homologs in *D*. *pteronyssinus airmid*. (DOCX)

S3 Table Top 10 most abundant proteins identified in *D*. *pteronyssinus airmid* mite body. (DOCX)

S4 Table Top 10 most abundant proteins identified in *D*. *pteronyssinus airmid* spent culture medium. (DOCX)

S1 Data Allergen orthologs potentially cross-reactive orthologs of known mite group allergens (Der p1-11, 13–16, 18, 20–33 or non-chronalogical allergen sequences available in UniProt for *D*. *pteronyssinus* and *D*. *farinae*, where *D*. *pteronyssinus* sequences were unavailable) identified by BLAST searches in 12 acari and 2 arachnid out-grouping. To be considered an allergen ortholog, proteins had to have reciprocal best hits with an allergen (E-value ≤1E-05), an alignment length of at least 80 amino acids and share at least 35% identity. We also identified allergen homologs by removing the criteria of being a reciprocal best hit.
(XLSX)

S2 Data Number of potentially cross-reactive orthologs of known MGA (Der p1-11, 13–16, 18, 20–33 or non-chronalogical allergen sequences available in UniProt for *D*. *pteronyssinus* and *D*. *farinae*, where *D*. *pteronyssinus* sequences were unavailable) identified by BLAST searches in 12 acari and two arachnid outgrouping. To be considered an allergen ortholog, proteins had to have reciprocal best hits with an allergen (E-value ≤1E-05), an alignment length of at least 80 amino acids and share at least 35% identity.
(XLSX)

S3 Data *D*. *pteronyssinus* and *D*. *pteronyssinus airmid* specific genes. *D*. *pteronyssinus* specific genes (*n* = 1,850) are absent from other acari but present in all one or more *D*. *pteronyssinus* genome assemblies (S1 Table). *D*. *pteronyssinus airmid* specific genes (*n* = 1,475) were found only in *D*. *pteronyssinus airmid* genome assembly.
(XLSX)

S4 Data *D*. *pteronyssinus airmid* predicted proteome (*n* = 12,530 sequences) annotated by blast homology to sequences in NCBInr, SwissProt and InterPro databases utilising Blast2GO. (XLSX)

S5 Data BLAST alignment results for *D*. *pteronyssinus airmid* predicted proteins (E-value ≤1E-03) to MGA sequences available in UniProt for *D*. *pteronyssinus* and *D*. *farinae* (where *D*. *pteronyssinus* sequences were unavailable). (XLSX)

S6 Data List of all *D*. *pteronyssinus airmid* protein sequences with known immunomodulatory effects or predicted, “allergenic molecules (*n* = 332)”, based upon BLASThomology to known allergens (E-value ≤1E-05), with an alignment length of at least 80 amino acids and share at least 35% identity. This list is a consolidation of protein sequences designated as“Mite Group Allergens and Non-chronological allergens” (MGA, *n* = 37), “Mite group allergen homologs” (MGAH, *n* = 93), “Allergen Homologs” (AH, *n* = 326). Allergen Homolog BLASTp alignment data.
(XLSX)

S7 Data List of *D*. *pteronyssinus airmid* proteins (*n* = 3,931) identified by meta-proteomic analysis of 88Gb of spectra (99 LC-MS/MS files) representing the proteome available for analysis utilising extraction and analysis techniques employed during this study (Representative Proteome). (XLSX)

S8 Data Proteomic results of all *D*. *pteronyssinus airmid* proteins (*n* = 150) identified from house dust protein extracts by LC-MS/MS. (XLSX)

S9 Data A) Proteomic results of all D. pteronyssinus airmid proteins (n = 1,849) identified from the mite body through combined proteomic strategy of whole extract protein analysis and gel filtration protein extract fractionation, followed by LC-MS/MS. B) List of the most specific GO terms (n = 167) differentially represented between the representative proteome and mite body proteome (<0.05 FDR) illustrating sample enrichment for proteins associated with mite body processes. C) List of enzyme names (n = 2) differentially represented between the representative proteome and mite body proteome (<0.05 FDR) illustrating sample enrichment for enzymes associated with mite body processes. D) List of InterPro IDs (n = 57) differentially represented between the representative proteome and mite body proteome (<0.05 FDR) illustrating sample enrichment of proteins with functional domains associated with mite body processes.
(XLSX)

S10 Data A) Proteomic results of all D. pteronyssinus airmid proteins (n = 873) identified from the spent culture media (Excretome) through combined proteomic strategy of whole extract protein analysis and gel filtration protein extract fractionation, followed by LC-MS/MS. B) List of the most specific GO terms (n = 320) differentially represented between the representative proteome and Excretome (<0.05 FDR) illustrating selective extraction of proteins associated with the Excretome. C) List of enzyme names (n = 35) differentially represented between the representative proteome and Excretome (<0.05 FDR) illustrating selective extraction of certain groups of enzymes associated with the Excretome. D) List of InterPro IDs (n = 69) differentially represented between the representative proteome and Excretome (<0.05 FDR) illustrating selective extraction of proteins with functional domains associated with the excretome.
(XLSX)

S11 Data List of all *D*. *pteronyssinus airmid* proteins identified in this study by proteomics (*n* = 4, 002). (XLSX)
