# Supplementary material for: Proteome and allergenome of the European house dust mite Dermatophagoides pteronyssinus
Source: PLoS One. 2019 May 1;14(5):e0216171. doi: 10.1371/journal.pone.0216171 (PMC6493757; doi:10.1371/journal.pone.0216171)
Supplement: S1 Table — (DOCX) [file pone.0216171.s001.docx]

| **S1** **Table: Genome data utilised in our assessment of genomic completeness and phylogenetic analysis** | | |
| --- | --- | --- |
| **Species** | **Genome Reference** | **Suborder** |
| *Centruroides sculpturatus* |  | Arachnid outgroup |
| *Parasteatoda tepidariorum* |  | Arachnid outgroup |
| *Dermatophagoides farinae* | (2) | Acariformes |
| *Euroglyphus maynei* | (3) | Acariformes |
| *Sarcoptes scabiei* | (4) | Acariformes |
| *Tetranychus urticae* | (5) | Acariformes |
| *Psoroptes ovis* | (6) | Acariformes |
| *Ixodes scapularis* | (7) | Parasitiforme |
| *Galendromus occidentalis* | (8) | Parasitiforme |
| *Rhipicephalus microplus* | (9) | Parasitiforme |
| *Tropilaelaps mercedesae* | (10) | Parasitiforme |
| *Varroa destructor* | (11) | Parasitiforme |
| *Varroa jacobsoni* |  | Parasitiforme |
