# Supplementary material for: Proteome and allergenome of the European house dust mite Dermatophagoides pteronyssinus
Source: PLoS One. 2019 May 1;14(5):e0216171. doi: 10.1371/journal.pone.0216171 (PMC6493757; doi:10.1371/journal.pone.0216171)
Supplement: S2 Table — (DOCX) [file pone.0216171.s002.docx]

| **S2 Table. LEA Homologs in *D. pteronyssinus airmid* assigned based upon single best highest scoring Blast hit with known LEA protein.** | | | | | |
| --- | --- | --- | --- | --- | --- |
| **Query Sequence ID^A^** | **Sequence ID^B^** | **Description^C^** | **LEA Class (Best hit)^D^** | **% Identities** | **E-value** |
| AMR19109 | DERPT_G248 | UDP-glucose 4-epimerase | 99 | 22.508 | 8.95E-07 |
| JAC82215 | DERPT_G290 | Phospholipase ABHD3 | 99 | 29.551 | 7.23E-54 |
| NP_001256174 | DERPT_G404 | Laminin subunit gamma-1 | 6 | 21.64 | 8.74E-09 |
| AMR19109 | DERPT_G997 | Natural resistance-associated Macrophage 1 Short | 99 | 25.904 | 9.20E-09 |
| JAC82215 | DERPT_G3026 | Phospholipase ABHD3 | 99 | 28.198 | 1.43E-49 |
| AMR19109 | DERPT_G3148 | GDP-mannose 4,6-dehydratase | 99 | 24.671 | 4.28E-13 |
| XP_006488730 | DERPT_G4173 | F-box LRR-repeat 20 | 6 | 25.824 | 3.02E-07 |
| NP_851129 | DERPT_G4823 | LEA homology | 6 | 29.688 | 1.41E-08 |
| KCW52581 | DERPT_G4831 | Allergen Homolog (Hev b9, Sac Enolase, Alt a 11) Enolase | 6 | 55.708 | 8.0E-152 |
| AMR19109 | DERPT_G6082 | UDP-glucose 4-epimerase | 99 | 26.036 | 3.51E-14 |
| BAE92616 | DERPT_G6261 | Flagellar attachment zone 1 | 6 | 22.145 | 6.06E-09 |
| AMR19109 | DERPT_G7527 | 3 beta-hydroxysteroid dehydrogenase Delta 5-- | 99 | 26.829 | 6.14E-08 |
| LEA76_BRANA | DERPT_G8349 | Zonadhesin Flags: Precursor | 6 | 21.053 | 2.03E-06 |
| JAC82215 | DERPT_G8791 | Phospholipase ABHD3 | 99 | 28.909 | 1.27E-43 |
| AMR19109 | DERPT_G10968 | UDP-glucuronic acid decarboxylase 1 | 99 | 24.924 | 4.37E-19 |
| AMR19109 | DERPT_G11799 | Fatty acyl- reductase 1 | 99 | 25.595 | 6.23E-06 |
| KCW52581 | DERPT_G12026 | Der f enolase like Allergen; Allergen homolog (Bla g Enolase, Sal s 2, Bos D Enolase), Proposed Pan-Allergen: Enolase | 6 | 56.308 | 3.6E-173 |
| BAE92616 | DERPT_G12094 | Liver stage antigen | 6 | 23.642 | 8.14E-08 |
| Query Sequence ID^A^, from LEAP database (http://forge.info.univ-angers.fr/~gh/Leadb/index.php). Sequence ID^B^, *D. pteronyssinus airmid* protein sequence ID. Description^C^, annotations assigned by Blast2GO. LEA Class (Best hit)^D^ , single best Blast alignment. | | | | | |
