# Supplementary material for: Proteome and allergenome of the European house dust mite Dermatophagoides pteronyssinus
Source: PLoS One. 2019 May 1;14(5):e0216171. doi: 10.1371/journal.pone.0216171 (PMC6493757; doi:10.1371/journal.pone.0216171)
Supplement: S3 Table — (DOCX) [file pone.0216171.s003.docx]

| **S3 Table. Top 10 Highest Abundant Protein Identified in *D. pteronyssinus airmid* Mite Body** | | | | | | | | | |
| --- | --- | --- | --- | --- | --- | --- | --- | --- | --- |
| **Sequence IDs^A^** | **Description^B^** | **N: WE^C^** | **N: Fractions^D^** | **N: Peptides^E^** | **N: Unique peptides^F^** | **Sequence coverage [%]** | **Mol. Mass [kDa]** | **N: MS/MS count^G^** | **LFQ**  **Intensity^H^** |
| DERPT_G9820 | Der p14 Allergen; Proposed Sten-Allergen, Allergen Homolog (Sar s14, Eur m14, Der f14) | 4 | 20 | 261 | 261 | 88.8 | 191.4 | 4729 | 2.2E+12 |
| DERPT_G8046 | Myosin-7 | 4 | 20 | 297 | 297 | 76.2 | 249.0 | 3554 | 1.1E+12 |
| DERPT_G11558 | ATP synthase subunit | 4 | 20 | 47 | 47 | 79.6 | 56.5 | 1101 | 7.6E+11 |
| DERPT_G8792 | Der p 2 Allergen; Proposed Mon-Allergen, Allergen Homolog (Der f2, Eur m2, Der s2) | 4 | 20 | 20 | 20 | 76.7 | 15.9 | 419 | 6.7E+11 |
| DERPT_G8780 | Der f30 like allergen: Ferritin heavy chain | 4 | 20 | 27 | 27 | 99.4 | 20.8 | 572 | 6.0E+11 |
| DERPT_G8047 | Der p10 Allergen; Proposed Pan-Allergen, Allergen Homolog (Der p10, Aca s10, Pso o10, Sar s10, Blo t10) | 4 | 20 | 51 | 51 | 81.7 | 33.0 | 923 | 5.4E+11 |
| DERPT_G749 | Allergen Homolog (Sal s3, Thua3): Fructose-bisphosphate aldolase | 4 | 20 | 42 | 42 | 85.9 | 39.4 | 667 | 4.9E+11 |
| DERPT_G8381 | Der p11 Allergen; Proposed Sten-Allergen, Allergen Homolog (Ani s2, Blo t11, Der f11, Der p11) | 4 | 20 | 173 | 173 | 48.9 | 184.0 | 1822 | 4.7E+11 |
| DERPT_G5156 | Der f aldehyde dehydrogenase like allergen; Allergen homolog (Tyr p 35, Cla h3) | 4 | 20 | 69 | 68 | 95.9 | 54.1 | 840 | 4.3E+11 |
| DERPT_G3392 | Allergen homolog (Tri a34, Asp vGAPDH): Glyceraldehyde-3-phosphate dehydrogenase | 4 | 20 | 53 | 52 | 98.5 | 35.7 | 674 | 3.9E+11 |
| Sequence ID^A^, *D. pteronyssinus airmid* protein sequence ID. Description^B^, annotations assigned by Blast2GO. N: WE^C^, number of whole protein extract replicates in which specified protein was identified. N: Fractions^D^, number of gel filtration fractions in which specified protein was identified. N: WE^D^, number of whole protein extract replicates in which specified protein was identified. N: Peptides^E^, number of peptides identified by LC-MS/MS for specified protein. N: Unique peptides^F^, number of unique (not present in any other protein sequence in the predicted proteome) peptides identified by LC-MS/MS for specified protein. N: MS/MS count^G^, sum of peptides selected for ms/ms analysis. LFQ Intensity^H^, label free quantification intensity. (Software: Maxquant version 1.6.2.10, Perseus version 1.6.2.2). | | | | | | | | | |
