# Supplementary material for: Proteome and allergenome of the European house dust mite Dermatophagoides pteronyssinus
Source: PLoS One. 2019 May 1;14(5):e0216171. doi: 10.1371/journal.pone.0216171 (PMC6493757; doi:10.1371/journal.pone.0216171)
Supplement: S4 Table — (DOCX) [file pone.0216171.s004.docx]

| **S4 Table. Top 10 Most Abundant Proteins Identified in Spent Culture Medium of *D. pteronyssinus airmid*** | | | | | | | | | |
| --- | --- | --- | --- | --- | --- | --- | --- | --- | --- |
| **Sequence IDs^A^** | **Description^B^** | **N: WE^C^** | **N: Fractions^D^** | **N: Peptides^E^** | **N: Unique peptides^F^** | **Sequence coverage [%]** | **Mol. Mass [kDa]** | **N: MS/MS count^G^** | **LFQ**  **Intensity^H^** |
| DERPT_G8792 | Der p 2 Allergen; Proposed Mon-Allergen, Allergen Homolog (Der f2, Eur m2, Der s2) | 4 | 21 | 17 | 17 | 78.8 | 15.9 | 486 | 5.2E+11 |
| DERPT_G1283 | Der p 1 Allergen, Proposed Sten-Allergen, Allergen Homolog (Der f1, Der m1, Pso o1) | 4 | 21 | 35 | 35 | 41.3 | 96.0 | 1511 | 4.9E+11 |
| DERPT_G8859 | Der p3 Allergen, Proposed Sten-Allergen, Allergen Homolog (Der p3, Der f3, Eur m3, Blo t3, Tyr p3) | 4 | 21 | 30 | 30 | 67.4 | 28.1 | 510 | 4.4E+11 |
| DERPT_G9697 | Sucrase- intestinal, alpha-1,4-glucosidase activity | 4 | 21 | 106 | 102 | 61.7 | 202.1 | 1288 | 3.4E+11 |
| DERPT_G3350 | Der p15 Allergen | 4 | 15 | 52 | 52 | 27.1 | 183.4 | 706 | 3.0E+11 |
| DERPT_G7100 | Der p9 Allergen | 4 | 21 | 24 | 24 | 87.2 | 29.4 | 440 | 2.9E+11 |
| DERPT_G9187 | Der f6 like allergen; Allergen Homolog (Blo t6) | 4 | 21 | 29 | 29 | 14.4 | 228.1 | 468 | 2.6E+11 |
| DERPT_G10989 | kDa bacteriolytic enzyme | 4 | 21 | 10 | 10 | 37 | 30.9 | 302 | 1.6E+11 |
| DERPT_G9149 | Lysosomal alpha-mannosidase | 4 | 11 | 89 | 89 | 74.5 | 118.3 | 875 | 1.6E+11 |
| DERPT_G9820 | Der p14 Allergen; Proposed Sten-Allergen, Allergen Homolog (Sar s14, Eur m14, Der f14) | 4 | 20 | 183 | 183 | 78.5 | 191.4 | 1388 | 1.6E+11 |
| Sequence ID^A^, *D. pteronyssinus airmid* protein sequence ID. Description^B^, annotations assigned by Blast2GO. N: WE^C^, number of whole protein extract replicates in which specified protein was identified. N: Fractions^D^, number of gel filtration fractions in which specified protein was identified N: Peptides^E^, number of peptides identified by LC-MS/MS for specified protein. N: Unique peptides^F^, number of unique (not present in any other protein sequence in the predicted proteome) peptides identified by LC-MS/MS for specified protein. N: MS/MS count^G^, sum of peptides selected for ms/ms analysis. LFQ Intensity^H^, label free quantification intensity. (Software: Maxquant version 1.6.2.10, Perseus version 1.6.2.2) | | | | | | | | | |
